# Fluoride concentration in ground water and prevalence of dental fluorosis in Ethiopian Rift Valley: systematic review and meta-analysis

**DOI:** 10.1186/s12889-019-7646-8

**Published:** 2019-10-16

**Authors:** Habtamu Demelash, Abebe Beyene, Zewdu Abebe, Addisu Melese

**Affiliations:** 1 College of Health Sciences, Department of Public Health, Debre Tabor University, Debre Tabor, Ethiopia; 2 Institution of Health, Department of Environmental Health Science and Technology, Jimma University, Jimma, Ethiopia; 3 College of Natural and computational Sciences, Department of Chemistry, Debre Markos University, Debre Markos, Ethiopia; 4 College of Medicine and Health Sciences, Department of medical laboratory science, Bahir Dar University, Bahir Dar, Ethiopia

**Keywords:** Dental fluorosis, Fluoride, Concentration, Rift Valley, Ethiopia

## Abstract

**Background:**

The concentration of fluoride in ground drinking water greater than the world health organization standard value imposes a serious health, social and economic problem in developing countries. In the Ethiopian Rift Valley where deep wells are the major source of drinking water, high fluoride level is expected. Though many epidemiological studies on fluoride concentration and its adverse effects have been conducted in the region, the result is highly scattered and needs systematically summarized for better utilization.

**Objective:**

This research is aimed at estimating the pooled level of fluoride concentration in ground drinking water and the prevalence of dental fluorosis among Ethiopian rift valley residences.

**Methods:**

Cochrane library, MEDLINE/PubMed and Google scholar databases were searched for studies reporting the mean concentration of fluoride in ground water and prevalence of dental fluorosis in Ethiopian Rift valley. Search terms were identified by extracting key terms from reviews and selected relevant papers and review medical subject headings for relevant terms.

**Results:**

The mean fluoride level in ground water and the prevalence of dental fluorosis were pooled from eleven and nine primary studies conducted in Ethiopian Rift Valley respectively. The pooled mean level of fluoride in ground water therefore was 6.03 mg/l (95% CI; 4.72–7.72, *p* < 0.001) and the pooled prevalence of dental fluorosis among residents in Ethiopian rift valley was 32% (95% CI: 25, 39%, *p* < 0.001), 29% (95% CI: 22, 36%, p < 0.001) and 24% (95% CI: 17, 32%, p < 0.001 for mild, moderate and sever dental fluorosis respectively. The overall prevalence of dental fluorosis is 28% (95% CI, 24, 32%, p < 0.001).

**Conclusions:**

Though, the concentration level varies across different part of the rift valley region, still the level of fluoride in ground drinking water is greater than the WHO standard value (1.5 mg/l). Relatively high-level pooled prevalence of dental fluorosis was also seen in Ethiopian rift valley. Therefore, further studies covering the temperature, exposure time and other intake path ways with large sample size is recommended. Interventional projects should be implemented to decrease the concentration of fluoride in the ground drinking water source.

## Background

Fluoride is one of among chemicals that has been shown to cause significant effects in people through drinking-water [1, 2]. It could enter the human body through the ingestion of food, drinking water, inhalation and dermal contact. However, drinking water is the most important exposure pathway of fluoride, as about 75–90% of fluoride intake [2, 3]. At its low concentrations in drinking-water, fluoride has beneficial effects on teeth development, but excessive exposure (greater than the WHO guideline value of 1.5 mg/l), can give rise to a number of adverse effects [4–6]. The health effect ranges from mild dental fluorosis to crippling skeletal fluorosis as the level and period of exposure increases [4, 7]. Dental fluorosis is a developmental disturbance of dental enamel, caused by successive exposures to high level of fluoride during tooth development [2, 8].

Although, the world health organization has set the fluoride guideline limit of 1.5 mg/l in drinking water, Over 260 million people globally are drinking water from sources with high fluoride concentrations [9]. The ground water fluoride concentration is severe in countries within East African Rift Valley like Tanzania, Kenya and Ethiopia. The highest fluoride concentration reported 2800 mg/l in Lake Nakuru in Kenya [4]. Like other African countries situated in Rift Valley, fluoride is also a major health problem for communities using ground water sources in the Ethiopian Rift valley area [3, 10–12]. Nearly 8 million people in the Ethiopian Rift Valley are drinking from high fluoride water sources [7]. Long-term use of high fluoride drinking water is known to cause both dental and skeletal fluorosis observed in populations residing in the Rift valley [2, 13, 14]. Several studies in African countries, including Tanzania, Sudan, Nigeria and Kenya have found a high prevalence of dental fluorosis among populations that consume drinking water with high fluoride content [4, 13, 15]. High fluoride concentration in ground water resulted high prevalence of dental fluorosis in the Ethiopian Rift Valley [7, 11, 12] which has lifelong health impacts ranged from loss of teeth to debilitating pain [7, 16]. About 13 million people also thought to be at risk of developing fluorosis in this region [3].

Many researchers tried to investigate the mean concentration of fluoride in ground drinking water sources and prevalence of dental fluorosis in Ethiopian rift valley. But these studies are highly scattered and need systematically reviewed and summarized for better utilization. Therefore, this research work aimed at filling this gap through calculating the pooled mean level of fluoride concentration in ground drinking water sources and prevalence of dental fluorosis among Ethiopian rift valley residences.

### Objectives

The objective of this systematic review and meta-analysis is to determine the level of pooled mean fluoride concentration in ground drinking water sources and the pooled prevalence of dental fluorosis in Ethiopian rift valley.

## Methods and materials

### Study area

The Ethiopian sector of the East African Rift system extends for more than 1000 km in a north east-south west to north- south direction from the Afar depression, at Red Sea-Gulf of Aden junction, southwards to the Turkana depression. It is a long and narrow strip of low-lying plain land in between the highlands. It stretches from the north eastern part of the country to the southern border with Kenya, and divides the highland masses into two, the central and eastern highlands of Ethiopia [4]*.* Groundwater is an important source of drinking water and the dominant source for domestic supply in the region where surface waters are scarce and seasonal [17–19]. This area of the country usually associated with high level of fluoride in groundwater because of volcanic and basaltic rocks, which are more likely to release a high concentration of fluoride [10, 17, 20].

### Data sources and search strategy

**Fig. 1:**
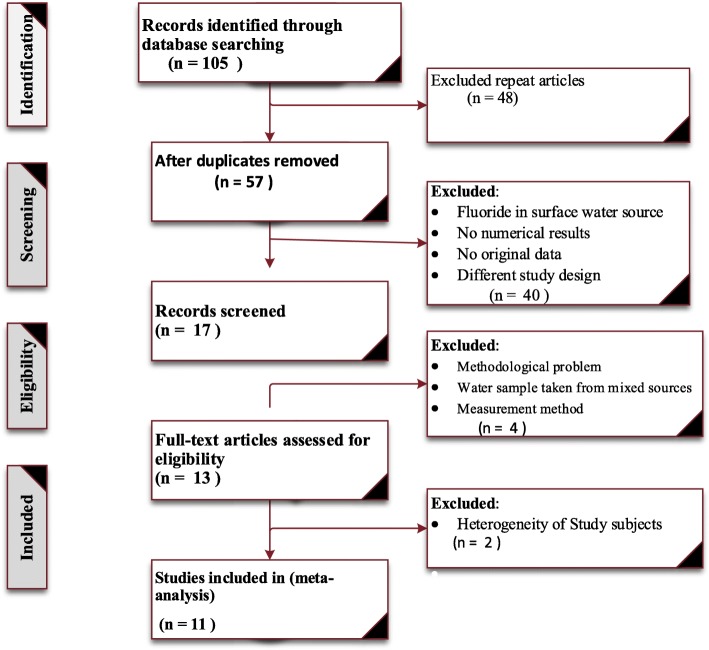
PRISMA flow chart of study selection

Peer reviewed journal articles which have reported the mean concentration of fluoride in ground water source and prevalence of dental fluorosis in Ethiopian Rift valley were searched via scientific databases, libraries and the journals themselves. Cochrane library, MEDLINE/PubMed and Google scholar databases were the main sources for the studies. The Preferred Reporting Items for Systematic Reviews and Meta-Analyses (PRISMA) statement guideline [21] was used to screen articles (Fig. 1). The following searching terms with Boolean operators were used to search PubMed; fluoride OR f-OR fluorosis OR drinking AND water OR ground AND water AND rift valley AND Ethiopia. In addition, we tried to check references of searched papers to obtain additional articles which is not identified in the databases.

### Inclusion and exclusion criteria

This review included all studies which measured the concentration of fluoride in ground drinking water sources and its outcome indicative (dental fluorosis) in Ethiopian rift valley region. Studies reported fluoride concentration (the minimum, maximum, mean and standard deviation in mg/l) were considered. In addition, the daily water consumption and fluoride intake per body weight also extracted from some studies. Regarding dental fluorosis, articles were selected based on fluorosis report with different severity level (mild, moderate and sever). Studies published in full text or abstract and published only in the English language (to avoid mistakes in the translation process) were included. Peer-reviewed and laboratory-based works with clear report of the result of laboratory tests also considered during screening (Table 1). All articles which did not meet the proposed criterion were excluded.

**Table 1:** Eligibility criteria

Inclusion criteria	Exclusion criteria
*− Country and setting*: Ethiopian rift valley	*−* The article did not contain original data or observations
*− Study design*: Cross-sectional	*−* Study subjects were not humans
*− Measurement*: The concentration of fluoride in ground water and prevalence of dental fluorosis	*−* Fluoride concentration was not measured or estimated
*− Types of article*: Peer-reviewed and published in English language	*−* Dental fluorosis was not reported using the three-severity level
*− Publication year*: up to December, 2018	*−* The mean and standard deviation of Fluoride concentration was not reported
*− Undertook laboratory works*: Reported type of laboratory tests and the respective result	*−* Being a review study
*− Measurable outcomes*: dental fluorosis (Dean’s index)	*−* Being none related to fluoride in ground water supplies and dental fluorosis

### Quality assessment

In order to assess the quality of included studies, the Newcastle-Ottawa Scale (NOS) which is adapted for cross-sectional studies (additional file 1) was used. Four investigators (HD, AB, ZA and AM) assessed the quality of included studies based on the efforts made by the authors of each primary studies to minimize the risk of error and bias. Inconsistencies between the quality assessor were discussed to reach consensus. But in most case the risk of bias for the prevalence of dental fluorosis across these studies was unjustifiable. To minimize the chance of error in data extraction, a pre-determined and standardized data extraction form was prepared and piloted with all review team members prior to the actual data extraction.

### Data extraction

Data extraction of all included papers was conducted by the three authors independently (HD, ZA and AM). These data extractors have taken practical training on the process of sourcing and recording relevant details from the primary studies included in the systematic review. Tailored Microsoft excel was used to ensure consistent data extraction, whilst reducing bias and improving validity. The following variables were extracted from studies: author, publication year, study population, water sample, number of human participants, age group and mean age of the participants, prevalence of dental fluorosis (mild, moderate and sever) and the concentrating of fluoride (minimum, maximum, mean and standard deviation in mg/l). But we didn’t contact study authors to obtain data needed for the analysis that were not reported in the published articles. The detail characteristics of included studies are shown in (Tables 2 and 3).

**Table 2:** Extraction for fluoride concentration of ground drinking waters in Ethiopian rift valley

Author Ref	[F^−^] in ground water (mg/l)			Daily Water Consumption (L/d)		F− intake (mg/kg bw/day)	
	Sample	Range	Mean + SD	Range	Mean + SD	Range	Mean + SD
T. Rango et al. [12], 2014	94	1.1–18	8.5 ± 4.1	0.33–2.7	1.2 ± 0.4	0.19–0.37	0.23 ± 0.2
T. Rango et al. [7], 2012	112	7.8–18	10 ± 40	0.5–3.0	1.2 + 0.34	0.15–0.60	0.36+ 0.18
T. Rango et al. [11], 2017	27	0.6–15	6.5 + 4.2	0.3–5.0	1.5+ 0.7	0.005–0.94	0.2 ± 0.16
Wondwossen F., et al. [14], 2004	30	0.3–14	6.6 + 3.13	–	–	–	–
Tekle-Haimanot R,. et.al [20], 2005	26	1.5–36	10.0 + 7.8	–	–	–	–
Tenalem Ayenew [22], 2008	51	0.1–75	4.94 + 1.16	–	–	–	–
Christopher J. et.al [23], 2018	47	4.8–5.2	5.0+ 3.45	–	–	–	–
Muhammed Haji,et al. [18], 2018	29	0.5–5.6	2.5 + 1.29	–	–	–	–
Tesfaw A, Feleke Z [10], 2011	16	3.2–4.19	4.16 + 2.31	0.25–3.21	1.6 + 0.17	0.25–0.31	0.28+ 0.03
Redda, Gebeyehu H [24], 2014	22	2.2–18.5	9.2 + 4.90	–	–	–	–
Aweke K.,et al. [19], 2016	60	2.6–6.5	4.6+ 1.90	–	–	–	–

**Table 3:** Summary of sensitivity analysis of the included studies

Excluded Studies	Pooled mean [F^−^] in mg/l (95% CI)	I^2^ (95% CI)	*P*-value
T. Rango et al.,2014 [22]	6.40 [4.70, 8.09]	97 [96,98]	< 0.001
T. Rangoa et al.,2012 [7]	6.16 [4.75, 7.58]	95 [93,97]	< 0.001
Aweke K.,et al., 2016 [19]	6.62 [4.98,8.27]	97 [96,98]	< 0.001
T. Rango et al.,2018 [11]	6.64 [4.88, 8.39]	97 [96,98]	< 0.001
Wondwossen et al.,2004 [14]	6.63 [4.85, 8.41]	97 [96,98]	< 0.001
Tekle-Haimanot R. et.al, 1987 [20]	6.33 [4.63, 8.03]	96 [94,97]	< 0.001
Tenalem Ayenew,2008 [22]	6.86 [4.61, 9.11]	97 [96,98]	< 0.001
Christopher J. et.al, 2018 [23]	6.82 [5.00, 8.63]	96 [94,97]	< 0.001
Muhammed Haji,et al.,2018 [18]	7.10 [5.50, 8.70]	97 [96,98]	< 0.001
Tesfaw A, Feleke Z,2011 [10]	6.91 [5.12, 8.69]	97 [96,98]	< 0.001
Redda, Gebeyehu H,2014 [24]	6.36 [4.65,8.07]	97 [96,98]	< 0.001

### Data analysis

All relevant studies which provided data on fluoride concentration in ground drinking water and crude prevalence of dental fluorosis or numbers of cases and study participants were included in the meta-analysis. Some studies reported the numerical concentration without calculating the mean fluorine concentration. In this case, the mean concentration of fluoride was calculated by summing up all concentration and divided by number of water samples. Whereas the prevalence of dental fluorosis for individual studies was determined by multiplying the ratio of dental fluorosis cases to sample size by 100.The estimation of pooled fluoride concentration and prevalence of dental fluorosis was done using CMA 2.0 and MetaXL version 5.3 software. With the assumption that true effect sizes exist between eligible studies, the random effects model was used to determine the pooled prevalence, mean fluoride and 95% CIs. A summary (pooled) estimate is calculated as a weighted average from selected studies as follows; weighted average = sum of (estimate X weight) sum of weights.

Heterogeneity was evaluated using the Cochran’s Q test and I^2^ statistics. Significant heterogeneity was declared at I^2^ > 50% and Q-test (*P* < 0.10).

### Publication bias and sensitivity analysis

The recurrence analysis under different assumptions to examine the impact of these assumptions on the results was done. Funnel plots were drawn to assess the possibility of publication bias and it indicated potential for publication bias. We plotted the studies’ logit event rate and the standard error to detect asymmetry in the distribution.

### Heterogeneity

The included eleven studies were assessed for heterogeneity. The differences between studies in the characteristics of their populations, in their study approaches and quality and the variation of effects between studies was checked using common sense, graphical and statistical tests. Forest plot was drawn and the overlapping confidence intervals was checked.

## Results

In this review, out of 105 searched articles, 11 articles which are completely relevant to the study objectives were finally selected and the required data were extracted. To determine fluorosis severity, Dean’s index was used in all studies. Water samples from ground water sources were taken at the frequency of 16 to 112 samples. The extracted data revealed that the distribution of fluoride in Ethiopian rift valley ground water varying widely ranged from 0.1 to 75 mg/l. The mean fluoride concentration above 1.5 mg/l have been reported from all included studies. The daily water consumption report was available from four studies which is ranged from 0.25 to 5.0 l/ day with the pooled mean daily water consumption of 1.366 l/day (95% CI; 1.15–1.58, *p* < 0.001). The results from daily Fluoride intake were also reported from four studies which is ranged from 0.005 to 0.94 mg/Kg bw/day with the pooled mean value of 0.271 mg/Kg bw/day (95% CI; 0.22–.0.33, p < 0.001) (Table 2).

### Sensitivity analysis

In this review, the sensitivity of each study was checked with the aim of identifying smaller or larger mean concentration reports which could affect the pooled result by giving wider confidence intervals and variance instability. But, the sensitivity analysis of this review showed no study has significantly affect the prime determinants of the pooled result (Table 3).

### Pooled mean fluoride concentration

**Fig. 2:**
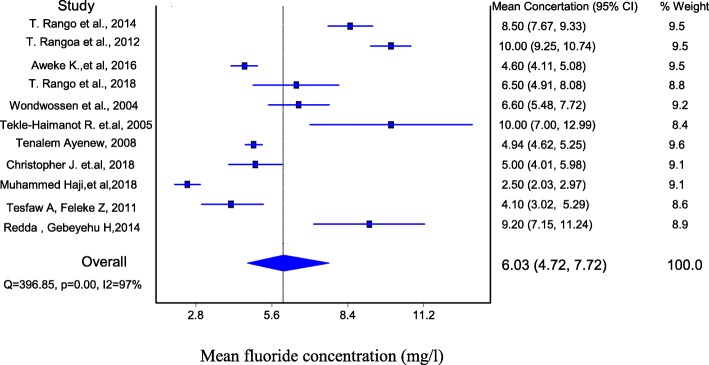
Forest plot of the pooled mean concentration of fluoride in ground water of Ethiopian rift valley

The data from eleven studies with total of 514 ground water samples in different part of Ethiopian rift valley were combined in this study. From the review, it was found that the mean fluoride concentration of the studies ranged from 2.5 to 10 mg/l with a substantial heterogeneity across studies (Q = 492.43; *p* < 0.001; I^2^ = 98%; 95% CI = 97.93–98.84). Meta-analysis was performed based on the mean and standard deviation of the concentration of fluoride in ground drinking water resources. Since the heterogeneity was > 50%, the random effect model was used to evaluate pooled fluoride concentration. The overall pooled mean concentration of fluoride in ground water of Ethiopian rift valley from random effects method was 6.03 mg/l (95% CI; 4.72–7.72, p < 0.001) which is above the WHO maximum allowable value of 1.5 mg/L (Fig. 2). Sensitivity analysis revealed no significant difference both in the pooled fluoride concentration and heterogeneity. When one study was excluded from the analysis step-by-step, the pooled mean concentration was between 6.16 and 7.10 mg/l (Table 3).

### Pooled prevalence of dental fluorosis

Nine articles were selected for meta-analysis of dental fluorosis. Two articles were excluded from meta-analysis because of not reporting the prevalence and reporting of overall prevalence rather than based on different severity level. Most of the reports from primary studies were reported the prevalence of dental fluorosis as mild, moderate and sever dental fluorosis separately. This meta-analysis also made the pooled prevalence estimation with the consideration of the mild, moderate, sever and overall dental fluorosis.

The effect of other variables (exposure time, exposure to fluoride in diet and air) on the prevalence of dental fluorosis was examined in the primary studies. The temperature was also considered as a confounding factor, which has been included in a few studies.

**Fig. 3:**
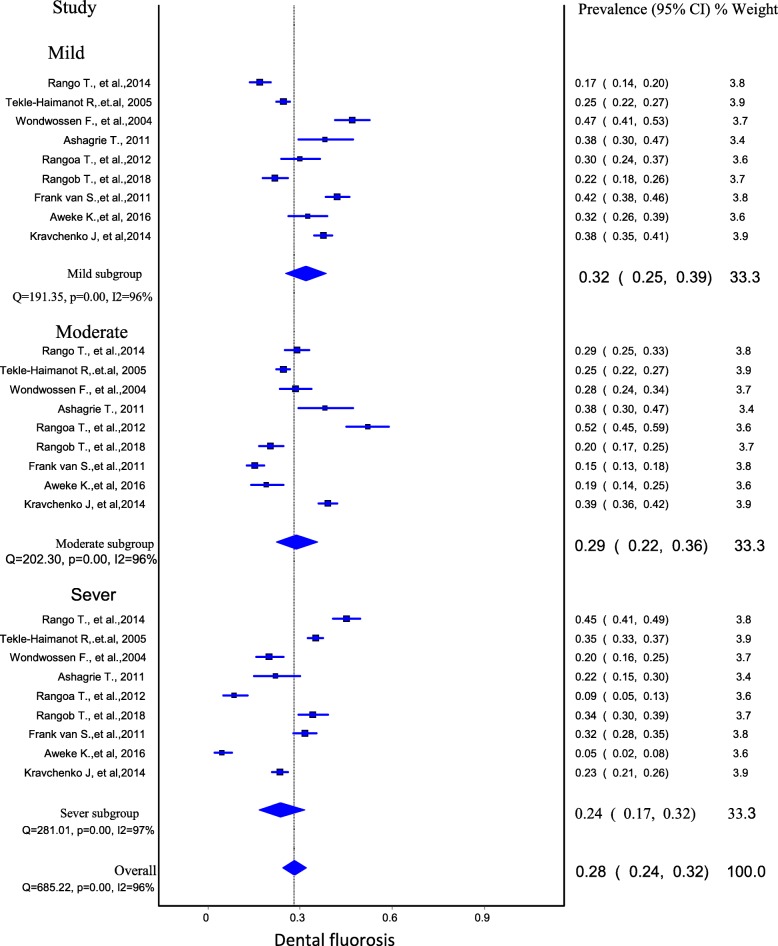
Forest plot of the prevalence of dental fluorosis among Ethiopian rift valley residents

A total of 4852 residents were participated in the study in the age range of 7 to 50-year-old (Table 4). The pooled prevalence of dental fluorosis among residents in Ethiopian rift valley was estimated to be 32% (95% CI: 25, 39%, *p* < 0.001) for mild dental fluorosis, 29% (95% CI: 22, 36%, p < 0.001) for moderate dental fluorosis and 24% (95% CI: 17, 32%, p < 0.001 for sever dental fluorosis. The overall prevalence of dental fluorosis is 28% (95% CI: 24, 32%, *p* < 0.001) (Fig. 3).

**Table 4:** Extraction for prevalence of dental fluorosis based on its severity among the understudy samples in Ethiopian rift valley

Author Ref	Sample	Age range	Mean ± SD	Prevalence of dental fluorosis		
				Mild	Moderate	Sever
T. Rango et al. [12], 2014	491	10–15	12.1 ± 1.6	17	29	45.00
T. Rango et al. [7], 2012	200	7–40	16.0 ± 6.2	30	52	8.40
T. Rango et al. [11], 2018	386	10–50	24.5 ± 11.1	21.7	20.4	34.10
WondwossenF.,et al. [14], 2004	306	12–15	13.5 ± 2.1	47	28	20.00
Tekle-Haimanot et.al [20], 2005	1456	7–20	13.2 ± 2.4	28	21	35.00
Frank van S et al. [25], 2011	625	10–15	13.2 ± 1.8	42	15.36	5.00
Tesfaw A, Feleke Z [10], 2011	118	10–15	12.1 ± 1.5	27.9	38.1	22.00
Aweke K.,et al. [19], 2016	216	10–15	12.4 ± 1.7	32.1	18.87	4.47
Kravchenko J, et al. [26], 2014	1054	10–30	13.1 ± 1.9	37.6	39	23.40

During the sensitivity analysis, when one study was excluded from the analysis step-by-step, the pooled prevalence of dental fluorosis was between 35 and 41%, for mild, 30 and 35% for moderate and 25 and 33% for sever dental fluorosis prevalence.

## Discussion

From this review, it was estimated that the mean fluoride concentration in ground drinking water of Ethiopian rift valley is high above the WHO standard (1.5 mg/l). Such elevated fluoride in drinking water sources gives the picture of severity of fluoride problem in Ethiopian rift valley with varies health risks associated with high fluoride concentration.

According to the research report in India [27], the mean concentration of fluoride in ground drinking water was lower than Ethiopian rift valley mean value. Similarly, other studies on fluoride content in ground drinking water in Iran [28, 29], revealed lower mean concentration than the fluoride concentration in Ethiopian rift valley ground water. The difference might be the effect of the east African rift valley which is known with active volcanic eruption resulting fluoride release and availability of different water sources for drinking in another part of the world.

Study reports from Kenyan Rift Valley revealed fluoride concentration level of 11 mg/L which is greater than this research result and Tanzanian rift valley, showed the fluoride concentration levels of 4.6 mg/L which is less than the pooled mean concentration of this research [30, 31]. According to a systematic review and metal analysis conducted in other African countries, the fluoride concentration in Uganda, South Africa, Gahna, Nigeria and Tanzania was 0.74 mg/L,1.27 mg/L, 2.09 mg/l,1.44 mg/l and 5.08 mg/l respectively [4]. All fluoride level in ground water source of the above African countries was lower than this pooled mean concentration of fluoride in Ethiopian rift valley except the mean concentration value in Kenya. The difference might be due to sample size and sampling techniques of the primary research and the characteristics of the geographical location of the region.

Though, the concentration level varies across different part of the rift valley region, still the level of fluoride in ground drinking water is greater than the WHO standard value. Other important measurements of fluoride exposure like the estimated daily fluoride intake per day and fluoride intake per body weight per day are more informative to assess the risk of fluoride concentration in ground water. But these are not discussed in detail in this report due to absence and incomplete report from primary studies.

The pooled prevalence of dental fluorosis among 4852 Ethiopian rift valley residences was estimated to be 28% ranging from 24 to 32%. Studies reported high prevalence of dental fluorosis in the Rift Valley region of Ethiopia where the level of fluoride, especially in groundwater is significantly high [14, 17, 20]. Evidences from other studies also indicated that dental fluorosis is well recognized health problem [2, 4, 8, 32]. According to the systematic review conducted in India, the pooled estimation of dental fluorosis was reported as 12.9% [33], which is much more lower than this result. Another study in Iran revealed the prevalence of dental fluorosis was 61% [34],which is more than the pooled estimation of this review.

According to a systematic review and meta-analysis in Iran, the pooled prevalence of dental fluorosis was 52.6% [8]. The study conducted in Tanzanian sector of the east African rift valley among 1434 children in the age range of 12–18 years, the prevalence of dental fluorosis was reported as 85.3% for mild, 75% for moderate and 41.4% for sever fluorosis [31] which is significantly greater than this review result.

The reported prevalence of dental fluorosis among 1549 participants in Ethiopian rift valley was estimated to be 70% for mild and 48.2% for sever [3]. The potential difference in the prevalence of dental fluorosis from the current pooled value might be due to the fact that weighted value for meta-analysis is likely affected by different factor from primary studies and application of different interventions in the area.

## Conclusion

This is the first systematic review and meta-analysis of the literature on the level of fluoride concentration in ground water and the prevalence of dental fluorosis in Ethiopian sector of East African rift valley. It is found that the pooled mean concentration of fluoride in ground drinking water and the pooled prevalence of dental fluorosis among residents in Ethiopian rift valley is expectedly high. Because the East African Rift Valley which cuts through Ethiopia is geomorphologically still an active volcanic region. It is also supported with other evidences from different part of East African rift valley. Since East African Rift Valley is still an active volcanic region, fluoride concentration is expected at high level in ground water for the future. Therefore, to address the health impact of high fluoride concentration in ground drinking water source of Ethiopian rift valley, use of alternative sources of drinking water as well as appropriate defluoridation technique is recommended. In addition, educating the community on dangers of using water with excess fluoride is recommended in order to ensure good health. The effort to establish long term trend of fluoride concentration in water should also be taken in consideration. This helps to predict future concentration of fluoride and possibility to minimize future risks. Comparative studies across different part of the rift valley to identify the priority area for intervention will helpful.

### Limitations

Only cross-sectional studies were included which are providing only snapshots of the situation at a particular moment in time and fail to capture the seasonal nature of the fluoride concentration in ground water. The number of primary studies for estimation of the pooled mean fluoride concentration and dental fluorosis were decreased (only eleven studies). In addition, this study was based only on published peer-reviewed in English language studies and important data might be missed from unpublished and published in other language studies and grey publications.

## Data Availability

All data generated or analyzed are included in the results of the manuscript.

## Abbreviations

CI: Confidence interval
ISE: Ion selective electrode
L: Liter
Mg: Milligram
TF: Thylstrup and Fejerskov
